# Tertiary lymphoid structures favor outcome in resected esophageal squamous cell carcinoma

**DOI:** 10.1002/cjp2.281

**Published:** 2022-06-16

**Authors:** Rutao Li, Xing Huang, Wenmin Yang, Jifan Wang, Yingkuan Liang, Te Zhang, Qixing Mao, Wenjie Xia, Lin Xu, Xinyu Xu, Gaochao Dong, Feng Jiang

**Affiliations:** ^1^ Department of Thoracic Surgery Nanjing Medical University Affiliated Cancer Hospital & Jiangsu Cancer Hospital & Jiangsu Institute of Cancer Research Nanjing PR China; ^2^ Jiangsu Key Laboratory of Molecular and Translational Cancer Research Cancer Institute of Jiangsu Province Nanjing PR China; ^3^ The Fourth Clinical College of Nanjing Medical University Nanjing PR China; ^4^ Department of Pathology, Jiangsu Cancer Hospital, Jiangsu Institute of Cancer Research The Affiliated Cancer Hospital of Nanjing Medical University Nanjing PR China; ^5^ Collaborative Innovation Center for Cancer Personalized Medicine Nanjing Medical University Nanjing PR China

**Keywords:** tertiary lymphoid structure, esophageal squamous cell carcinoma, prognostic value, immunotherapy target

## Abstract

Tertiary lymphoid structures (TLSs) are considered to have a good prognosis in multiple solid tumors. However, the prognostic value of TLS in esophageal squamous cell carcinoma (ESCC) is unknown. In this study, we retrospectively enrolled 185 ESCC patients who underwent surgical resection. Hematoxylin and eosin staining was performed to investigate the presence, the abundance, the maturation, and the location of TLSs. We explored the cellular composition of TLSs using traditional immunohistochemistry in serial sections. The prognostic value of TLSs was investigated by univariate and multivariate analyses. A nomogram was constructed to predict the prognosis. TLS‐positive tumors were infiltrated with more CD45^+^ leukocytes, CD20^+^ B cells, CD4^+^ and CD8^+^ T cells, and CD11c^+^ dendritic cells（DCs） compared with negative tumors. Kaplan–Meier curves showed that the presence and the abundance of TLSs were associated with longer disease‐free survival (DFS) (*p* = 0.0130) and overall survival (OS) (*p* = 0.0164). In addition, patients with tumors containing more CD20^+^ B cell infiltration had longer DFS (*p* = 0.0105) and OS (*p* = 0.0341). Multivariate analyses demonstrated that the presence of TLSs was an independent prognostic factor for DFS (hazard ratio [HR] = 0.384, *p* < 0.001) and OS (HR = 0.293, *p* < 0.001). The nomogram that integrated the tumor stage, histologic grade, and TLS presence had higher prognostic accuracy. Our study suggests that ESCC‐related TLSs can be used as a new biomarker for the prognosis of ESCC patients, and further understanding of their formation and mechanism of induction can provide a possible direction and target for immunotherapy of ESCC.

## Introduction

Esophageal cancer is the seventh most common cancer and sixth leading cause of cancer‐related mortality worldwide; the major histologic subtype is esophageal squamous cell carcinoma (ESCC), which has a particularly high incidence in Eastern Asia and in Eastern and Southern Africa [1]. Clinical outcomes of ESCC remain poor, despite advances in surgical treatments over the past few decades [2]. Recently, patients with advanced or metastatic ESCC who received camrelizumab (an anti‐programmed death receptor 1 [PD‐1] antibody) added to chemotherapy were shown to have significantly improved overall survival (OS) and progression‐free survival, compared with placebo and chemotherapy [3], which revolutionized the treatment of ESCC. The success of immunotherapy in ESCC underscores the need to better understand the tumor microenvironment that leads to effective anti‐tumor response. T cells have been considered the major immune cells able to induce tumor regression; however, recent publications focused on tertiary lymphoid structures (TLSs) have indicated that they may also play a prominent role in immunotherapy [4].

TLSs are ectopic lymphoid structures that develop in inflamed tissues, such as in chronic infections, autoimmune diseases, and cancers, and are important elements of the tumor‐immune microenvironment [5]. In addition to T cells, TLSs are mainly composed of B cells and follicular dendritic cells (FDCs), fibroblast reticular cells, stromal cells, dendritic cells, neutrophils, macrophages, and endothelial cells. FDCs are usually located in the center of B cells and express various receptors that facilitate antigen presentation to B cells; they also promote the expression of highly active antibodies by B cells [6]. The composition of TLSs can synergistically improve the immune response of the body and promote the killing of tumors, but some of the high‐depletion molecules, such as PDL1, Treg cells, and myeloid‐derived suppressor cells, can also inhibit immunity to varying degree. TLSs predict favorable prognosis in most solid cancers, including pancreatic cancer [7], colorectal cancer [8], and gastrointestinal stromal tumors [9]. However, there were very few studies on ESCC addressing the prognostic and predictive values of TLS and it is not clear whether TLSs could furnish additional value in predicting the survival of ESCC patients.

In this study, we evaluated the presence, abundance, maturation, and location of TLSs in a cohort of 185 patients with primary ESCC treated by surgical resection. These were randomly divided into a discovery cohort with 122 patients and a validation cohort with 63 patients. Here, we aimed to study the prognostic significance of TLSs in ESCC and explore their association with tumor‐infiltrating immune cells in this tumor type.

## Materials and methods

### Patients and samples

A total of 185 patients who underwent surgical resection and histopathologic diagnosis for ESCC at The Affiliated Cancer Hospital of Nanjing Medical University between January 2017 and December 2018 were included. The patients' inclusion criteria were as follows: (1) pathologically diagnosed as primary ESCC; (2) no chemotherapy or radiotherapy performed before surgery; (3) sufficient preservation of the paraffin‐fixed tissue, with clear structure; and (4) the presence of a complete medical treatment record and follow‐up information, with a follow‐up time longer than 2 years. For all patients, pathologic stages were defined according to the eighth edition of the American Joint Committee on Cancer (AJCC) cancer staging manual using the available clinical and pathologic tumor, node, and metastasis data. Patient information, including age and gender, and clinicopathologic characteristics, such as tumor size and histologic grade, were also collected. Disease‐free survival (DFS) was defined as the time from the date of surgery to the date of recurrence. OS was defined as the time from the date of surgery to the date of either death or the last follow‐up. All patients were randomly divided into a discovery cohort (*n* = 122) and a validation cohort (*n* = 63). The study was approved by the Ethics Committee of The Affiliated Cancer Hospital of Nanjing Medical University. Samples were obtained from biobank of Jiangsu Cancer Hospital (Jiangsu Institute of Cancer Research & The Affiliated Cancer Hospital of Nanjing Medical University). All patients had signed informed consent for donating their samples.

### H&E staining

The paraffin‐embedded human ESCC tissue specimens were cut into 5‐μm serial sections. The sections were dewaxed and rehydrated with xylene and ethanol, respectively, followed by staining with hematoxylin for 5 min and 1% acid ethanol for 3 s. The sections were then rinsed in distilled water and stained with eosin for 3 min. Dehydration and hyalinization were then subsequently performed. Sections were visualized using a light microscope (Nikon, Tokyo, Japan).

### IHC staining

The formalin‐fixed paraffin‐embedded ESCC tissue specimens were cut as serial 5‐μm sections. The sections were deparaffinized in xylene and hydrated through an ethanol series. The sections were then boiled for 5 min in citrate buffer (pH 6.0) and left to cool in order to retrieve epitopes. After quenching endogenous peroxidase with 3% hydrogen peroxide for 15 min, nonspecific binding was blocked by incubating the sections in 10% horse serum, followed by incubation with primary antibodies at 4 °C overnight. The sections were then rinsed and incubated with appropriate biotinylated secondary antibodies for 30 min at 37 °C, and then rinsed again and incubated with peroxidase‐conjugated streptavidin for 30 min at 37 °C. Finally, the sections were reacted with diaminobenzidine solution for 5 min, followed by counterstaining with hematoxylin. The sections were viewed and photographed with a light microscope (Nikon). Immunohistochemistry (IHC) was performed to detect CD45, CD20, CD4, CD8, CD11c, and CD68 expression in patient samples using a standard protocol. The antibodies and conditions used in IHC are summarized in supplementary material, Table S1.

### Evaluation of TLS and IHC

**Figure 1:**
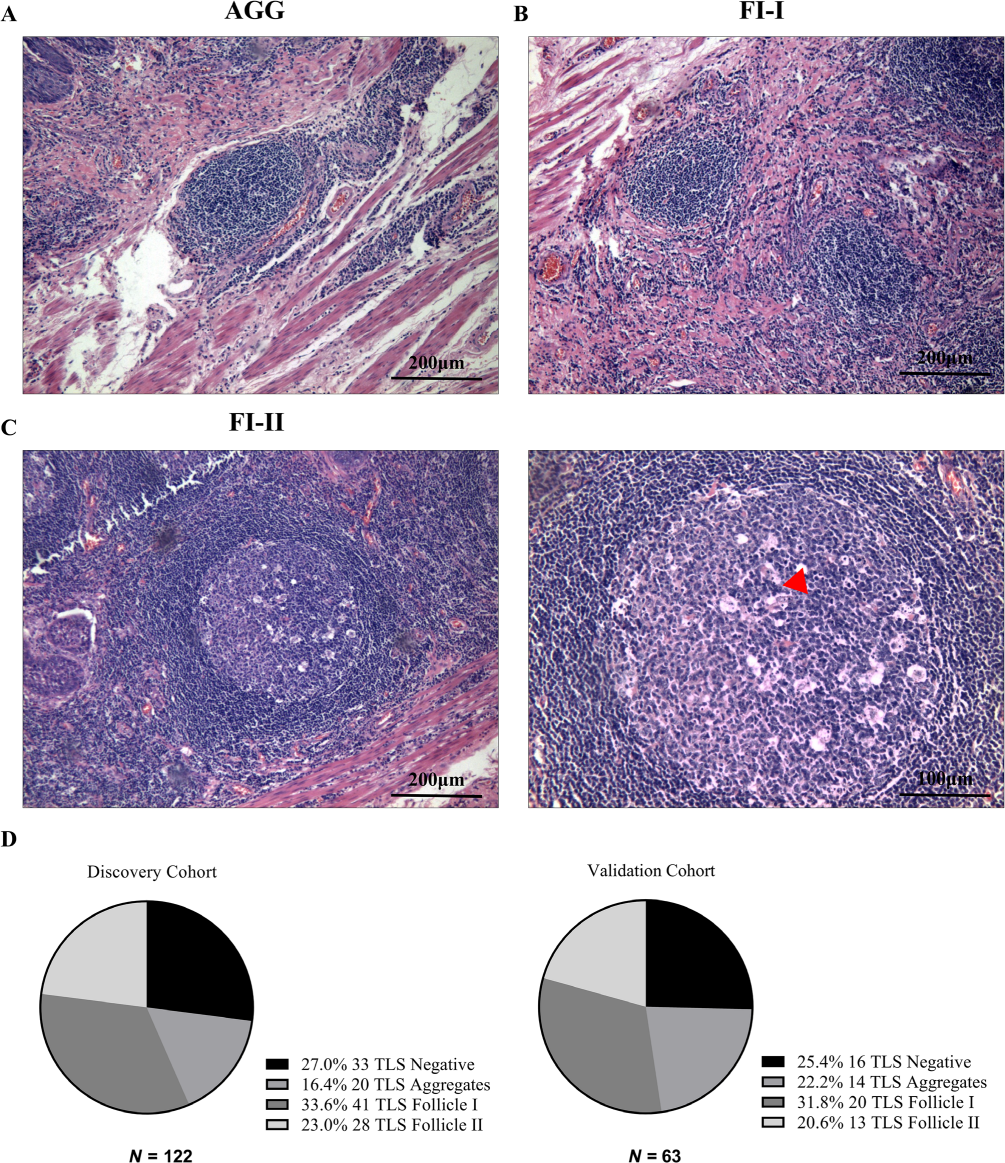
The classification of TLS in ESCC. (A) Representative H&E image of TLS Aggregates patients. (B) Representative H&E image of TLS Follicles I patients. (C) Representative H&E image of TLS Follicles II patients (red arrow: germinal center). (D) TLS phenotype and frequency evaluated in discovery (left) and validation cohorts (right). Proportions of TLS Negative, TLS Aggregates, Follicle I, and Follicle II in ESCC were summarized by pie chart.

**Figure 2:**
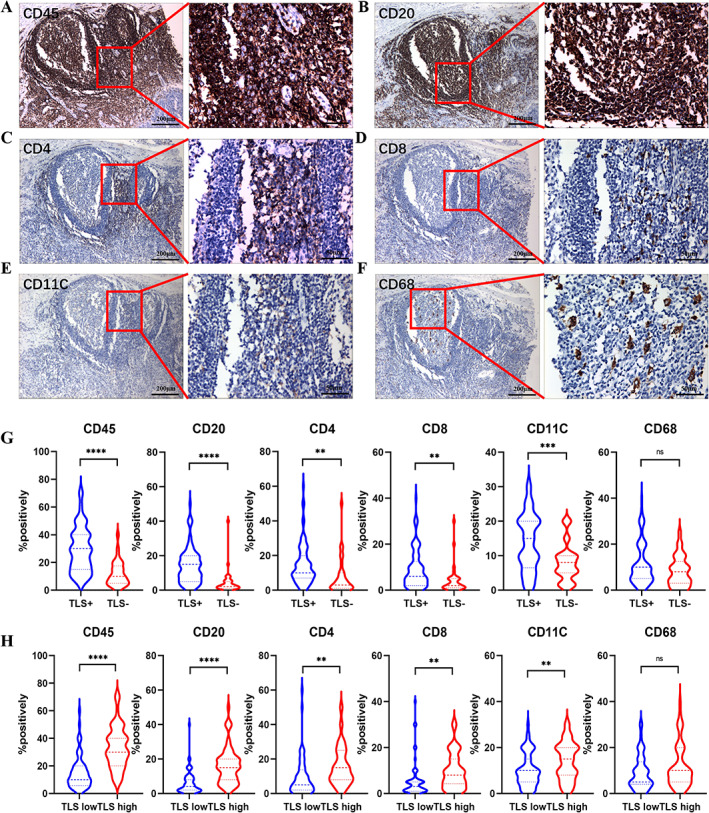
Serial sections of immune cells composition in TLSs. (A–F) Representative IHC image of cellular composition of TLS. (A) CD45^+^ lymphocytes. (B) CD20^+^ B cells. (C) CD4^+^ T cells. (D) CD8^+^ T cells. (E) CD11c^+^ DCs. (F) CD68^+^ TAMs. Different immune infiltration cells between TLS‐positive and ‐negative patients (G) and between TLS‐low and ‐high patients (H).

TLSs are organized aggregates of immune cells that form postnatally in non‐lymphoid tissues and arise in the context of chronic inflammation, such as in autoimmune disease, chronic infection, and cancer [10]. On hematoxylin and eosin (H&E)‐stained slides, TLSs were identified morphologically as distinct ovoid lymphocytic aggregates containing high endothelial venules and/or a germinal center [11]. The presence, abundance, location, and subtypes of TLS were assessed on H&E‐stained slides, which were reviewed by two independent pathologists without any clinical information about the patient. Where the assessment was controversial, a third pathological reviewer was involved in a discussion to resolve differences. Before screening the H&E‐stained slides, the two pathologists agreed on the definition and classification of TLSs, and most of the results were consistent. TLSs were evaluated on whole H&E sections in all the available tumor blocks of each patient. The maximum number of TLSs was selected to define the TLS abundance of the patient [12]. After all TLSs were marked by the pathologist, Xing Huang and Jifan Wang recorded and counted all TLSs in the entire pathological section. After counting and analyzing the TLS abundance of all patients, we defined the TLS‐high group as the abundance of TLS more than median 4 and TLS‐low group as less than or equal to median 4 [13]. Among the tumors with more than one TLS, we used the maximum number of mature TLSs to categorize the tumor. We defined the location with the greatest number of TLSs as the location of TLSs for this patient. We first performed H&E staining to assess the presence of TLSs in ESCC specimens. Then, TLSs observed around the tumor tissue or in the peritumoral area were defined as peritumoral TLS (supplementary material, Figure S1A) and TLSs within the tumor tissue were defined as intratumoral TLS (supplementary material, Figure S1B). Tumor‐infiltrating TLS were divided into three categories according to the morphology determined by H&E staining; (1) TLS aggregates (AGG): small, quasi‐circular clusters of lymphocytes (Figure 1A); (2) TLS follicles 1 (FL‐I): large clusters without germinal center formation (Figure 1B); and (3) TLS follicles 2 (FL‐II): large clusters with germinal center formation (Figure 1C). In serial sections, the percentage of staining area of each immune cell type in the entire pathological section was used as the degree of infiltration of the immune cell for subsequent analysis.

### Statistical analyses

All statistical analyses were performed using SPSS version 26 (SPSS, Chicago, IL, USA) and GraphPad Prism 8 (GraphPad Software Inc., La Jolla, CA, USA). The Wilcoxon signed‐rank test, Mann–Whitney test, Spearman correlation, and log‐rank test were used, as appropriate. Multivariate analyses were performed using Cox proportional hazards regression to identify independent prognostic factors. Kaplan–Meier analysis was used to compare differences after curative surgery for patient DFS and OS. All tests were two‐sided and were performed with a significance level of *α* = 0.05.

## Results

### Patient characteristics and TLS status in ESCC

In this study, we retrospectively enrolled 185 patients who met the inclusion criteria and divided them randomly into two cohorts; 122 patients were included in the discovery cohort and the other 63 patients in the validation cohort. The detailed clinical characteristics of these two patient cohorts are summarized in Table 1, and no obvious differences were found in the clinical characteristics between the two cohorts. We evaluated the presence and maturation of TLSs (defined in Materials and methods section) and identified them in 89 patients (73.0%) in the discovery cohort. Among patients with TLSs, the maximum degree of TLS maturation was AGG, FL‐I, and FL‐II in 20 (16.4%), 41 (33.6%), and 28 (23.0%) cases, respectively (Figure 1D, left). In the validation cohort, 47 patients (74.6%) were considered positive with TLS, and the distribution of TLS maturation was similar to that in the discovery cohort, among which the maximum degree was 14 (22.2%), 20 (31.8%), and 13 (20.6%) patients with AGG, FL‐I, and FL‐II, respectively (Figure 1D, right). The location of TLSs (defined in Materials and methods section) was also evaluated. Among positive patients in the discovery cohort, 20 (16.4%) and 69 (56.6%) cases contained intratumoral and peritumoral TLSs, respectively (supplementary material, Figure S1C, left); in the validation cohort, 13 (20.6%) and 34 (54.0%) cases were intratumoral and peritumoral, respectively (supplementary material, Figure S1C, right).

**Table 1:** Clinicopathologic characteristics of patients in discovery and validation cohorts

Variables	Discovery cohort (*n* = 122)	Validation cohort (*n* = 63)	*P* value
	No. (%)	No. (%)	
Gender			0.651
Male	102 (83.61)	51 (80.95)	
Female	20 (16.39)	12 (19.05)	
Age (years)			0.635
≤60	35 (28.36)	16 (25.40)	
>60	87 (71.31)	47 (74.60)	
Smoke			0.477
Smoking	59 (48.36)	27 (42.86)	
Non‐smoking	63 (51.64)	36 (57.14)	
Drink			0.553
Drinking	52 (42.62)	24 (38.10)	
Non‐drinking	70 (57.38)	39 (61.90)	
Site			0.111
Upper	8 (6.56)	10 (15.88)	
Middle	59 (48.36)	25 (39.68)	
Lower	55 (45.08)	28 (44.44)	
Size (mm)			0.361
≤40	57 (46.72)	25 (39.68)	
>40	65 (53.28)	38 (60.32)	
Histologic grade			0.177
1	19 (15.58)	12 (19.05)	
2	49 (40.16)	32 (50.79)	
3	54 (44.26)	19 (30.16)	
Lymph node			0.256
Yes	91 (74.59)	42 (66.67)	
No	31 (25.41)	32 (33.33)	
Tumor stage			0.082
I	10 (8.20)	5 (7.94)	
II	36 (29.51)	30 (47.62)	
III	71 (58.20)	25 (39.68)	
IV	5 (4.10)	3 (4.76)	
pT			0.728
T1	23 (18.85)	12 (19.05)	
T2	25 (20.49)	16 (25.40)	
T3	74 (60.66)	35 (55.56)	
pN			0.038
N0	33 (27.05)	29 (46.03)	
N1	56 (45.90)	17 (26.98)	
N2	28 (22.95)	14 (22.22)	
N3	5 (4.10)	3 (4.76)	
Perineural invasion			0.711
Yes	28 (22.95)	16 (25.40)	
No	94 (77.05)	47 (74.60)	
Lymphovascular space invasion			0.976
Yes	39 (31.97)	20 (31.75)	
No	83 (68.03)	43 (68.25)	
TLS			0.634
TLS+	89 (72.95)	48 (76.19)	
TLS−	33 (27.05)	15 (23.81)	
Recurrence			0.675
Yes	58 (47.54)	32 (50.79)	
No	64 (52.46)	31 (49.21)	
Death			0.959
Yes	45 (36.89)	23 (36.51)	
No	77 (63.11)	40 (63.49)	

### Correlations between TLSs and tumor‐infiltrating immune cells in ESCC patients

To better understand the relationship between TLSs and the tumor microenvironment, we explored the general composition of TLSs [6] in ESCC patients using traditional IHC in serial sections stained for CD45^+^ lymphocytes, CD20^+^ B cells, CD4^+^ T cells, CD8^+^ T cells, and CD11c^+^ DCs. We found that CD20^+^ B cells tended to be located in the center of the follicle, whereas CD4^+^ T cells, CD8^+^ T cells, and CD11c^+^ DCs were primarily located in the parafollicular zone (Figure 2A–E), indicating that the cellular composition of TLSs in ESCC is consistent with previous results reported in other tumors [14]. Interestingly, CD68^+^ tumor‐associated macrophages (TAMs), which suppress or activate immune responses, were distributed in the center of the follicle [4] (Figure 2F). We also compared the prominent immune subsets between different TLS classifications and showed that tumors containing TLSs were infiltrated with more CD45^+^ leukocytes, CD20^+^ B cells, CD4^+^ and CD8^+^ T cells, and CD11c^+^ DCs compared with tumors without TLSs. We also evaluated the CD68^+^ TAMs, and there was no observed significant difference among the two groups (Figure 2G). The same results can be seen in TLS‐high/low groups (Figure 2H). We also investigated the effect of different maturation of TLSs on immune cell infiltration. However, there was no significant difference in the infiltration of the six immune cells in three types of TLS (supplementary material, Figure S2A). Additionally, we explored the effect of TLS location on infiltrating immune cells. We found increased infiltration of CD8+ T cells in peritumoral TLSs than in intratumoral TLSs (supplementary material, Figure S2B). Taken together, these results reveal that tumors positive for TLSs and with higher numbers of TLSs were infiltrated with more adaptive immune cells, the major components of TLSs.

### Correlations between TLSs and clinicopathologic characteristics in ESCC patients

After screening the components of TLSs in ESCC, we further explored the correlations between TLSs and clinicopathologic characteristics (Table 2). There was no correlation between TLSs and gender, age, smoking, drinking, tumor size, tumor site, lymphovascular space invasion, perineural invasion, or histologic grade (*p* > 0.05) in both the discovery and validation cohorts. The presence of TLSs correlated positively with lower recurrence (discovery cohort, *p* < 0.001; validation cohort, *p* = 0.045) and relatively well‐defined favorable survival (discovery cohort, *p* < 0.001; validation cohort, *p* = 0.030). Interestingly, patients with high numbers of TLSs also had lower recurrence (discovery cohort, *p* = 0.019; validation cohort, *p* = 0.032) and death than those with low TLSs in both cohorts (discovery cohort, *p* = 0.003; validation cohort, *p* = 0.018).

**Table 2:** The correlation between TLS and clinicopathologic features in the whole series (*n* = 185)

	Discovery cohort (*n* = 122)						Validation cohort (*n* = 63)					
Variables	TLS+	TLS−	*P* value	TLS low	TLS high	*P* value	TLS+	TLS−	*P* value	TLS low	TLS high	*P* value
Gender			0.185			0.683			0.914			0.646
Male	72 (80.90)	30 (90.91)		51 (85.00)	51 (82.26)		39 (81.25)	12 (80.00)		26 (78.79)	25 (83.33)	
Female	17 (19.10)	3 (0.09)		9 (15.00)	11 (12.74)		9 (18.75)	3 (20.00)		7 (21.21)	5 (16.67)	
Age (years)			0.810			0.474			0.219			0.168
≤60	25 (28.09)	10 (30.30)		19 (31.67)	16 (25.81)		14 (29.17)	2 (13.33)		6 (18.18)	10 (33.33)	
>60	64 (71.91)	23 (69.70)		41 (68.33)	46 (74.19)		34 (70.83)	13 (86.67)		27 (81.82)	20 (66.67)	
Smoke			0.671			0.722			0.393			0.275
Smoking	42 (47.19)	17 (51.52)		30 (50.00)	29 (46.77)		22 (45.83)	5 (33.33)		12 (36.36)	15 (50.00)	
Non‐smoking	47 (52.81)	16 (48.48)		30 (50.00)	33 (53.23)		26 (54.17)	10 (66.67)		21 (63.64)	15 (50.00)	
Drink			0.700			0.191			0.098			0.064
Drinking	37 (41.57)	15 (45.45)		22 (36.67)	30 (48.39)		21 (43.75)	3 (20.00)		9 (27.27)	15 (50.00)	
Non‐drinking	52 (58.43)	18 (54.55)		38 (63.33)	32 (51.61)		27 (56.25)	12 (80.00)		24 (72.72)	15 (50.00)	
Site			0.372			0.778			0.730			0.089
Upper	7 (7.87)	1 (3.03)		3 (5.00)	5 (8.06)		8 (16.67)	2 (13.33)		4 (12.12)	6 (20.00)	
Middle	40 (44.94)	19 (57.58)		29 (48.33)	30 (48.39)		20 (41.67)	5 (33.33)		10 (30.30)	15 (50.00)	
Lower	42 (47.19)	13 (39.39)		28 (46.67)	27 (42.55)		20 (41.67)	8 (53.33)		19 (57.58)	9 (30.00)	
Size (mm)			0.864			0.461			0.565			0.572
≤40	42 (47.19)	15 (45.45)		26 (43.33)	31 (50.00)		20 (41.67)	5 (33.33)		12 (36.36)	13 (43.33)	
>40	47 (52.81)	18 (54.55)		34 (56.67)	31 (50.00)		28 (58.33)	10 (66.67)		21 (63.64)	17 (56.57)	
Histologic grade			0.336			0.631			0.180			0.338
1	14 (15.73)	5 (15.15)		8 (13.33)	11 (17.74)		11 (22.92)	1 (6.67)		4 (12.12)	8 (26.67)	
2	39 (43.82)	10 (30.30)		23 (38.33)	26 (41.94)		25 (52.08)	7 (46.67)		18 (54.55)	14 (46.67)	
3	36 (40.45)	18 (54.55)		29 (48.33)	25 (40.32)		12 (52.00)	7 (46.67)		11 (33.33)	8 (26.67)	
Lymph node metastasis			0.264			0.350			1.000			0.593
Yes	64 (71.91)	27 (81.82)		47 (78.33)	44 (70.97)		32 (66.67)	10 (66.67)		21 (63.64)	21 (70.00)	
No	25 (28.09)	6 (18.18)		13 (21.67)	18 (29.03)		16 (33.33)	5 (33.33)		12 (36.36)	9 (30.00)	
Tumor stage			0.061			0.327			0.188			0.407
I and II	38 (42.70)	8 (24.24)		20 (33.33)	26 (41.94)		22 (45.83)	4 (26.67)		12 (36.36)	14 (46.67)	
III and IV	51 (57.30)	25 (75.76)		40 (66.67)	36 (58.06)		26 (54.17)	11 (73.77)		21 (63.64)	16 (53.33)	
Perineural invasion			0.240			0.596			0.418			0.720
Yes	18 (20.22)	10 (30.30)		15 (25.00)	13 (20.97)		11 (22.92)	5 (33.33)		9 (27.27)	7 (23.33)	
No	71 (79.78)	23 (69.70)		45 (75.00)	49 (79.03)		37 (77.08)	10 (66.67)		24 (72.73)	23 (76.67)	
Lymphovascular space invasion			0.284			0.274			0.628			0.777
Yes	26 (29.21)	13 (39.39)		22 (36.67)	17 (27.42)		16 (33.33)	4 (26.67)		11 (33.33)	9 (30.00)	
No	63 (70.79)	20 (60.61)		38 (63.33)	45 (72.58)		32 (66.67)	11 (73.33)		22 (66.67)	21 (70.00)	
Recurrence			<0.001			0.019			0.045			0.032
Yes	33 (37.08)	25 (75.76)		35 (58.33)	23 (37.10)		21 (43.75)	11 (73.33)		21 (63.64)	11 (36.67)	
No	56 (62.92)	8 (24.24)		25 (41.47)	39 (62.90)		27 (56.25)	4 (26.67)		12 (36.36)	19 (63.33)	
Death			<0.001			0.003			0.030			0.018
Yes	25 (28.09)	21 (63.64)		30 (50.00)	15 (24.19)		14 (29.17)	9 (60.00)		16 (48.48)	6 (16.17)	
No	64 (71.91)	12 (36.36)		30 (50.00)	47 (75.81)		34 (70.83)	6 (40.00)		17 (51.52)	25 (83.33)	

### Correlations between TLS and survival in ESCC patients

**Figure 3:**
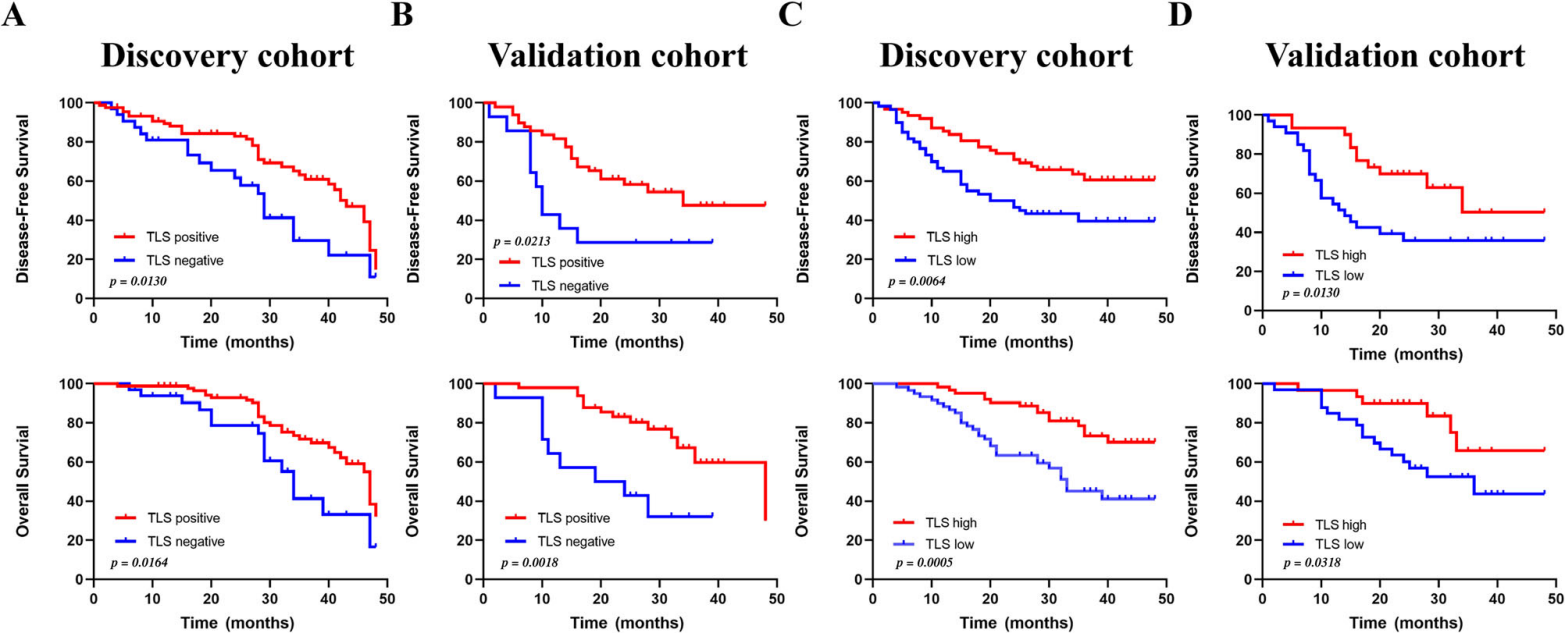
Prognostic significance of TLS presence and abundance in ESCC patients. (A, B) Kaplan–Meier analysis of DFS and OS in discovery cohort (*n* = 122) (A) and validation cohort (*n* = 63) (B) according to TLS presence. (C, D) Kaplan–Meier analysis of DFS and OS in discovery cohort (*n* = 122) (C) and validation cohort (*n* = 63) (D) according to TLS abundance.

We explored the prognostic value of TLSs by performing Kaplan–Meier curves in ESCC patients. The median follow‐up time was 29 months (range: 1–48 months). During the follow‐up period, 42.6% (58 of 122) of patients relapsed and 36.9% (45 of 122) died in the discovery cohort. We observed that TLS^+^ patients had better OS (discovery cohort, *p* = 0.0164; validation cohort, *p* = 0.0018) and DFS (discovery cohort, *p* = 0.0130; validation cohort, *p* = 0.0213) compared to TLS^−^ patients (Figure 3A,B). ESCC patients in the TLS‐high group had longer DFS (discovery cohort, *p* < 0.01; validation cohort, *p* < 0.05) and OS (discovery cohort, *p* < 0.001; validation cohort, *p* < 0.05) compared to patients in the TLS‐low group (Figure 3C,D).

**Figure 4:**
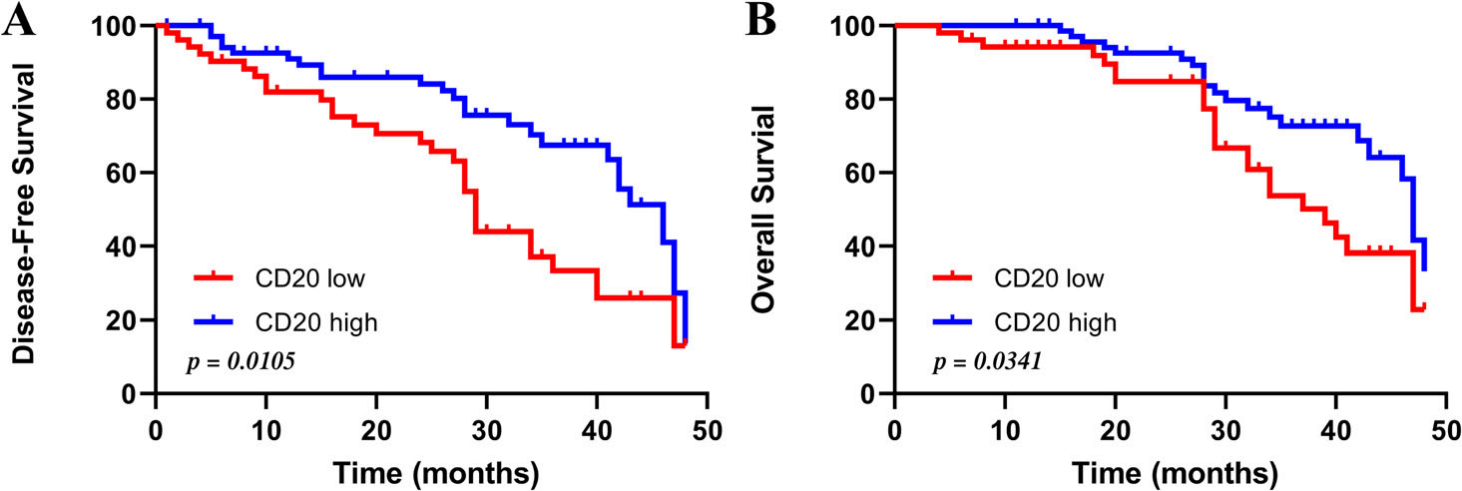
CD20^+^ B cell infiltration favors a good prognosis in ESCC patients. (A, B) Kaplan–Meier analysis of DFS (A) and OS (B) in discovery cohort (*n* = 122) according to CD20^+^ B cell infiltration.

We then explored the impact of TLS maturation on prognosis. We found no significant differences in DFS and OS according to TLS phenotype in both discovery and validation cohorts (supplementary material, Figure S3A,B). This may be due to the relatively small sample size of our cohort. We further explored whether prominent immune cells of TLSs could have an impact on the prognosis of ESCC patients. Survival analysis was performed according to the IHC score of each immune cell, which was divided into two groups based on the median of levels of pre‐stained immune infiltrations. Only patients with more CD20^+^ B cell infiltration had longer DFS (*p* = 0.0105) and OS (*p* = 0.0341) (Figure 4). We consider that B cells, as the main components of TLS, contribute the main function [15].

In addition, univariate and multivariate analyses of clinicopathologic features was performed on the whole series as shown in Table 3. Lymph node metastasis (discovery cohort, hazard ratio [HR] = 2.695, *p* = 0.009; validation cohort, HR = 2.829, *p* = 0.022), tumor stage (discovery cohort, HR = 4.485, *p* < 0.001; validation cohort, HR = 5.847, *p* < 0.001), abundance (discovery cohort, HR = 0.491, *p* = 0.008; validation cohort, HR = 0.420, *p* = 0.02), and presence (discovery cohort, HR = 0.325, *p* < 0.001; validation cohort, HR = 0.418, *p* = 0.02) of TLSs were associated with DFS in ESCC. Multivariate Cox regression analyses identified tumor stage (discovery cohort, HR = 0.234, *p* < 0.001; validation cohort, HR = 0.281, *p* = 0.005) and TLS presence (discovery cohort, HR = 0.384, *p* < 0.001; validation cohort, HR = 0.444, *p* = 0.032) as independent prognostic factors for DFS.

**Table 3:** Univariate and multivariate analyses of clinicopathologic features, DFS, and OS in the whole series (*n* = 185)

	Discovery cohort (*n* = 122)				Validation cohort (*n* = 63)			
	DFS		OS		DFS		OS	
Variables	HR (95% CI)	*P* value	HR (95% CI)	*P* value	HR (95% CI)	*P* value	HR (95% CI)	*P* value
Univariate analysis								
Gender (male versus female)	1.366 (0.467–2.882)	0.413	1.928 (0.687–6.416)	0.213	0.970 (0.399–2.360)	0.947	1.296 (0.478–3.515)	0.61
Age (years) (≤60 versus >60)	1.120 (0.647–1.938)	0.686	1.598 (0.763–3.349)	0.214	1.722 (0.706–4.196)	0.232	1.617 (0.545–4.798)	0.387
Smoke (smoking versus non‐smoking)	1.135 (0.678–1.900)	0.629	1.077 (0.583–1.987)	0.814	1.017 (0.505–2.046)	0.963	1.868 (0.760–4.589)	0.173
Drink (drinking versus non‐drinking)	1.139 (0.680–1.909)	0.62	1.211 (0.650–2.257)	0.547	1.248 (0.601–2.592)	0.552	1.880 (0.734–4.815)	0.188
Site (upper versus middle versus lower)	2.158 (0.514–9.063)	0.293	1.267 (0.294–5.463)	0.751	1.287 (0.415–3.995)	0.662	0.980 (0.253–3.798)	0.977
	2.195 (0.523–9.233)	0.283	1.646 (0.385–7.029)	1.646	1.700 (0.568–5.090)	0.343	1.477 (0.415–5.529)	0.548
Size (mm) (≤40 versus >40)	1.652 (0.971–2.812)	0.064	1.180 (0.636–2.472)	0.6	1.385 (0.667–2.875)	0.382	1.219 (0.511–2.908)	0.656
Lymphovascular space invasion (yes versus no)	1.389 (0.812–2.373)	0.23	1.309 (0.692–2.472)	0.406	1.625 (0.730–3.621)	0.235	2.697 (0.908–8.006)	0.074
Perineural invasion (yes versus no)	1.694 (0.962–2.985)	0.068	1.610 (0.821–3.157)	0.166	1.063 (0.476–2.370)	0.882	1.043 (0.408–2.665)	0.931
Histologic grade (1 versus 2 versus 3)	3.053 (0.907–10.278)	0.072	3.702 (0.855–16.028)	0.08	4.215 (0.977–18.176)	0.054	2.281 (0.505–10.307)	0.284
	5.684 (1.744–18.524)	0.004	5.223 (1.239–22.014)	0.024	5.725 (1.277–25.654)	0.023	3.803 (0.819–17.656)	0.088
Lymph node metastasis (yes versus no)	2.695 (1.276–5.693)	0.009	2.293 (1.023–5.141)	0.44	2.829 (1.163–6.883)	0.022	2.875 (0.967–8.544)	0.057
Tumor stage (I and II versus III and IV)	4.485 (2.260–8.901)	<0.001	4.017 (1.865–8.652)	<0.001	5.847 (2.230–15.328)	<0.001	2.353 (1.205–4.596)	0.012
TLS (low versus high)	0.491 (0.289–0.832)	0.008	0.349 (0.187–0.652)	0.001	0.420 (0.201–0.874)	0.02	4.144 (1.392–12.331)	0.011
TLS (TLS+ versus TLS−)	0.325 (0.193–0.549)	<0.001	0.281 (0.156–0.506)	<0.001	0.418 (0.200–0.873)	0.02	0.302 (0.128–0.710)	0.006
Multivariate analysis								
TLS (TLS+ versus TLS−)	0.384 (0.225–0.657)	<0.001	0.293 (0.162–0.530)	<0.001	0.444 (0.212–0.931)	0.032	0.266 (0.112–0.634)	0.003
TLS (low versus high)	NA	0.985	NA	0.225	NA	0.165	NA	0.174
Tumor stage (I and II versus III and IV)	0.234 (0.116–0.473)	<0.001	0.260 (0.120–0.561)	0.001	0.281 (0.115–0.685)	0.005	NA	0.495
Lymph node metastasis (yes versus no)	NA	0.428	NA	NA	NA	0.086	3.297 (1.097–9.908)	0.034
Histologic grade (1 versus 2 versus 3)	2.137 (0.627–7.291)	0.225	NA	0.899	NA	0.561	NA	NA
	4.040 (1.223–13.345)	0.022	NA	0.205	NA	0.714	NA	NA

With regard to OS, univariate Cox regression analyses identified tumor stage (discovery cohort, HR = 4.017, *p* < 0.001; validation cohort, HR = 2.353, *p* = 0.012), TLS abundance (discovery cohort, HR = 0.349, *p* = 0.001; validation cohort, HR = 4.144, *p* = 0.015), and TLS presence (discovery cohort, HR = 0.281, *p* < 0.001; validation cohort, HR = 0.302, *p* = 0.006) as clinicopathologic factors that contributed to OS. Furthermore, multivariate analyses identified that only TLS presence (discovery cohort, HR = 0.293, *p* < 0.001; validation cohort, HR = 0.266, *p* = 0.003) was an independent indicator for OS. Taken together, these results suggested that the presence of TLS signified a favorable prognosis for ESCC.

**Figure 5:**
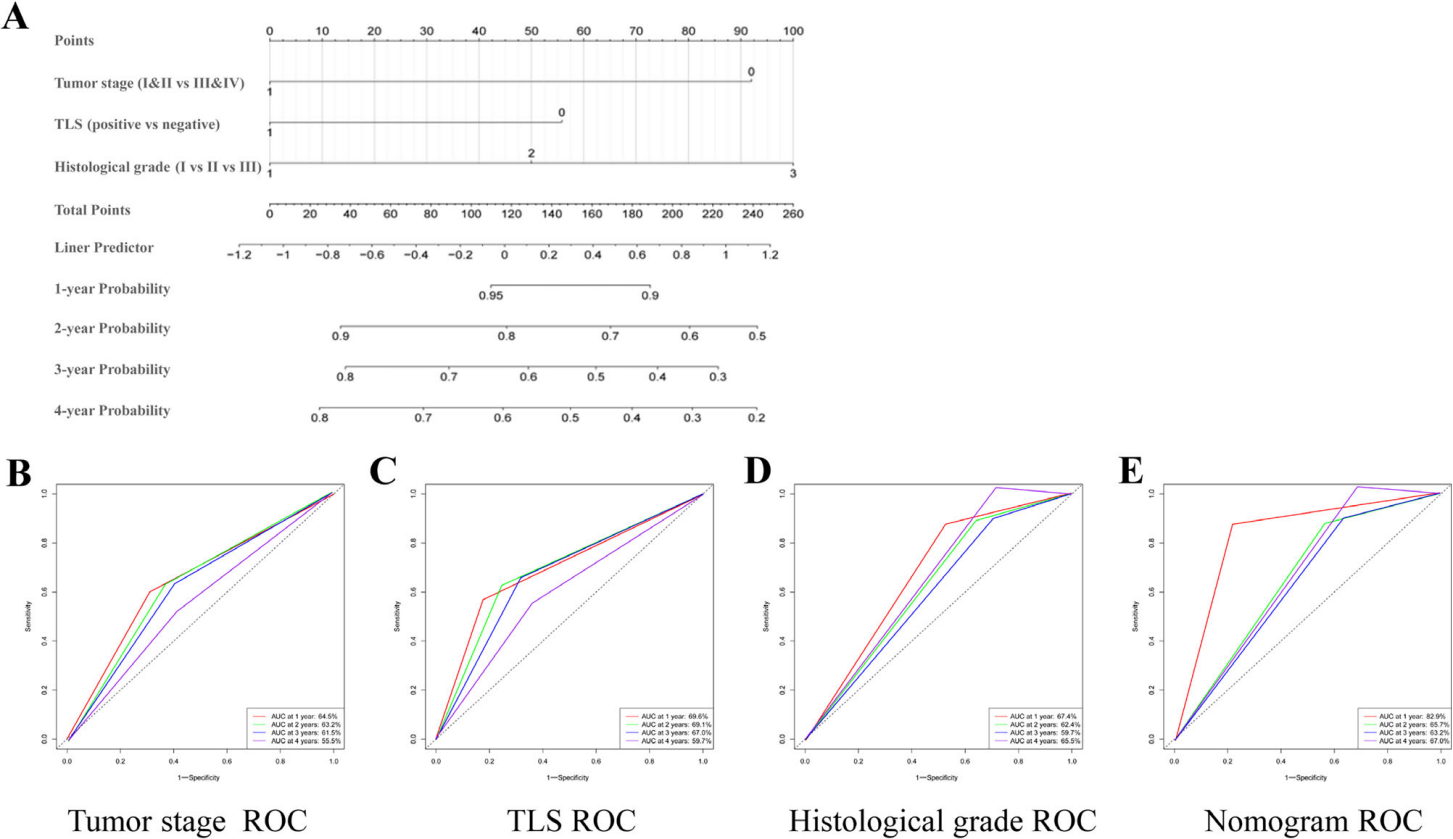
Nomogram predicting the probability of 1‐, 2‐, 3‐, 4‐year OS and TLS‐independent receiver operating characteristic (ROC) curve analyses in whole series. (A) Nomogram for predicting the probability of 1‐, 2‐, 3‐, and 4‐year OS for ESCC patients of the whole series. ROC curve analysis was used to compare the predictive accuracy between tumor stage (B), TLS presence (C), histologic grade (D), and nomogram (E) in 1, 2, 3, and 4 years.

Finally, we aimed to construct a nomogram, incorporating the presence of TLSs, to predict the probability of 1‐, 2‐, 3‐, and 4‐year OS, based on the multivariate Cox analyses. Then, a nomogram that integrated the tumor stage, histologic grade, and TLS presence was constructed to provide clinicians with a quantitative approach to predict the prognosis of patients with ESCC (Figure 5A). Receiver operating characteristic curve analysis was used to compare the predictive accuracy between nomogram, tumor stage, histologic grade, and TLS presence in 1, 2, 3, and 4 years (Figure 5B–E). The nomogram model suggested higher prognostic accuracy for 1‐, 2‐, 3‐, and 4‐year OS with a larger AUC.

## Discussion

In this study, we explored the relationship between the presence, the abundance, the maturation, and the location of TLSs and the outcomes of patients with resected ESCC. The presence of TLSs was an effective predictor of favorable prognosis for ESCC, as was the abundance of TLSs, confirmed using both discovery and validation cohorts. Additionally, multivariate analyses identified that only TLS presence was an independent indicator compared with TLS abundance. However, in our study, we found no correlation between the maturation or location of TLSs and the prognosis of ESCC patients. These findings provide new insights into the development of innovative prognostic markers for ESCC.

TLSs have been widely investigated by researchers and the effect of TLSs on the prognosis of many types of cancer has been reported, including lung cancer [16] and ovarian cancer [17], showing that patients with TLS‐positive tumors have longer DFS and OS. However, in addition to the presence of TLSs, their impact on prognosis is also affected by the tissue of origin, location, abundance, and maturation. For example, the presence of TLSs in colorectal cancer has been associated with more advanced disease [18]. Even in the same type of cancer, Gu‐Trantien *et al* found that CD4+ T cell immune infiltrates, principally located in TLS germinal centers, suggest a good prognosis by promoting anti‐tumor immunity in breast cancer [19], while TLSs in breast cancer were associated with higher tumor grade, low representation of intratumoral immune cells, and a high frequency of lymph node metastasis, as reported by Figenschau *et al* [20]. It is important to understand whether TLSs are formed by different mechanisms and play different roles in different cancer types for its application in predicting prognosis. In our study, we found no correlation between the location of TLSs and the prognosis of ESCC patients. However, Ding *et al* discovered that the abundance of intratumoral TLSs was an effective predictor of favorable prognosis for intrahepatic cholangiocarcinoma, while the presence of peritumoral TLSs was significantly associated with dismal outcomes [21]. The same results were found in hepatocellular carcinoma [22] and breast cancer [11]. TLS abundance was reported to signify a favorable prognosis in human oral cancer [12], which was consistent with our results. Finally, we found no difference in prognosis based on TLSs of different maturity, although the *P* value of survival analysis was very close to 0.05. Although it is not clear whether the maturity of TLSs affects patient outcomes, by analyzing the prognosis of colorectal cancer, Posch *et al* concluded that mature TLSs containing germinal centers had more prognostic value than immature TLSs, and believed that the determination of mature TLSs in colorectal cancer had more implications for good prognosis than TLS counting alone [23]. Mature TLSs are often accompanied by the formation of germinal centers, indicating active anti‐tumor immunity. Therefore, evaluation of TLS maturation in each tumor may be more accurate to determine the prognosis of cancer in the future.

To gain a better understanding of TLS in ESCC patients, we stained serial sections for the major components of TLSs. With regard to immune cell subtypes, CD45^+^ leukocytes, CD4^+^ T cells, CD8^+^ T cells, CD20^+^ B cells, and CD11c^+^ DCs accounted for the majority. We observed that ESCC patients with positive TLSs tended to be surrounded by more CD45^+^ leukocytes, as well as a higher number of tumor‐infiltrating CD4^+^ T cells, CD8^+^ T cells CD20^+^ B cells, and CD11c^+^ DCs. CD8^+^ T cells play a central role in the adaptive immune response in cancer. We found increased infiltration of CD8^+^ T cells in peritumoral TLSs than in intratumoral TLSs, suggesting that peritumoral TLSs may have better ability to inhibit tumor growth. However, Calderaro *et al* reported that intratumoral TLSs are associated with a low risk of early recurrence of hepatocellular carcinoma [22]. On the other hand, TLSs have been reported to promote the synergistic anti‐tumor effect of tumor‐associated plasma cells and CD8^+^ T cells [24]. Mature DCs can promote antigen presentation of T cells and B cells, and CD45^+^ lymphocytes participate in T cell signal transduction. Tumor‐infiltrating macrophages (CD68^+^ TAMs) are associated with tumor progression and low survival [18]. However, this study found no correlation between CD68^+^ TAMs and TLSs in tumors. Meanwhile, TLSs were mostly composed of a T cell zone in the outer layer and a B cell zone in the inner layer. CD11c^+^ DCs are present within the T cell zone, and B cells can cluster and form B cell follicles with actively replicating B cell germinal centers [25]. Then, TLSs promote effective anti‐tumor immune responses by promoting local antigen presentation and lymphocyte differentiation. Mature DCs provide antigen to T cells and activate cellular immunity in the T cell region, while germinal center LAMP^+^ DCs provide antigen to B cells and induce humoral immunity. Almost all TLSs contain B cells, and high expression of CD20^+^ B cells in the tumor microenvironment is associated with a good prognosis in most cancers such as biliary tract cancer [26] and skin cancer [27]. We also found that patients with more CD20^+^ B cell infiltration had longer DFS (*p* = 0.0105) and OS (*p* = 0.0341), indicating that B cell‐related pathways may play a key role in the generation and formation of TLSs.

Finally, we integrated tumor stage, histologic grade, and the presence of TLSs to provide clinicians with a new nomogram model that may be used to predict OS for resected ESCC patients. TLSs are located in or near tumor tissue and promote lymphocyte transport and infiltration, making them an interesting target for anti‐tumor immunity [28]. It is an interesting hypothesis to suggest that inducing TLS formation may also be an effective strategy for anti‐tumor immunity in ESCC, given the significant association between TLSs and better prognosis. To date, several therapies targeting TLSs have been developed and have shown promising anti‐tumor effects in various mouse models [29], and current cancer immune therapy such as checkpoint inhibitors may be more effective in combination with drugs that induce TLS generation. In the future, patients with immune resistance may benefit from the use of drugs inducing the formation or maturation of TLSs.

In conclusion, we have shown that the presence of TLSs is associated with longer DFS and OS, which is an independent favorable prognostic indicator for patients with ESCC undergoing surgical resection. Additionally, patients with more CD20^+^ B cell infiltration, the major component of TLSs, also have longer DFS and OS. This study reveals the correlation between TLS state and prognosis of patients with ESCC and provides a framework to better understand the components of TLSs, which provide an immune therapeutic avenue of clinical interest.

## Author contributions statement

RL, XH and WY contributed to sample and data acquisition and manuscript drafting. JW, YL, TZ, QM and WX offered technical support. LX, XX, GD and FJ made substantial contributions to the conception and design of the study, funding of the study, and supervision. All authors were involved in writing the paper and had final approval of the submitted and published versions.

## Supporting information


**Figure S1.** The location of TLSs in esophageal cancer
**Figure S2.** Association between maturation and location of TLSs and immune infiltration
**Figure S3.** Prognostic significance of TLS maturation in ESCC patients
**Table S1.** The antibodies and conditions used in IHC in this study
