# Supplementary material for: Tertiary lymphoid structures favor outcome in resected esophageal squamous cell carcinoma
Source: J Pathol Clin Res. 2022 Jun 16;8(5):422–35. doi: 10.1002/cjp2.281 (PMC9353661; doi:10.1002/cjp2.281)
Supplement: Supplementary file 1 — Figure S1. The location of TLSs in esophageal cancer Figure S2. Association between maturation and location of TLSs and immune infiltration Figure S3. Prognostic significance of TLS maturation in ESCC patients Table S1. The antibodies and conditions used in IHC in this study [file CJP2-8-422-s001.pdf]

Tertiary lymphoid structures favor outcome in resected esophageal squamous cell carcinoma

R Li, X Huang, W Yang *et al*, *J Pathol Clin Res,* DOI: 10.1002/cjp2.281

Supplementary Material

Figure S1. The location of tertiary lymphoid structures (TLSs) in esophageal cancer

Figure S2. Association between maturation and location of TLSs and immune infiltration

Figure S3. Prognostic significance of TLS maturation in ESCC patients

Table S1. The antibodies and conditions used in IHC in this study

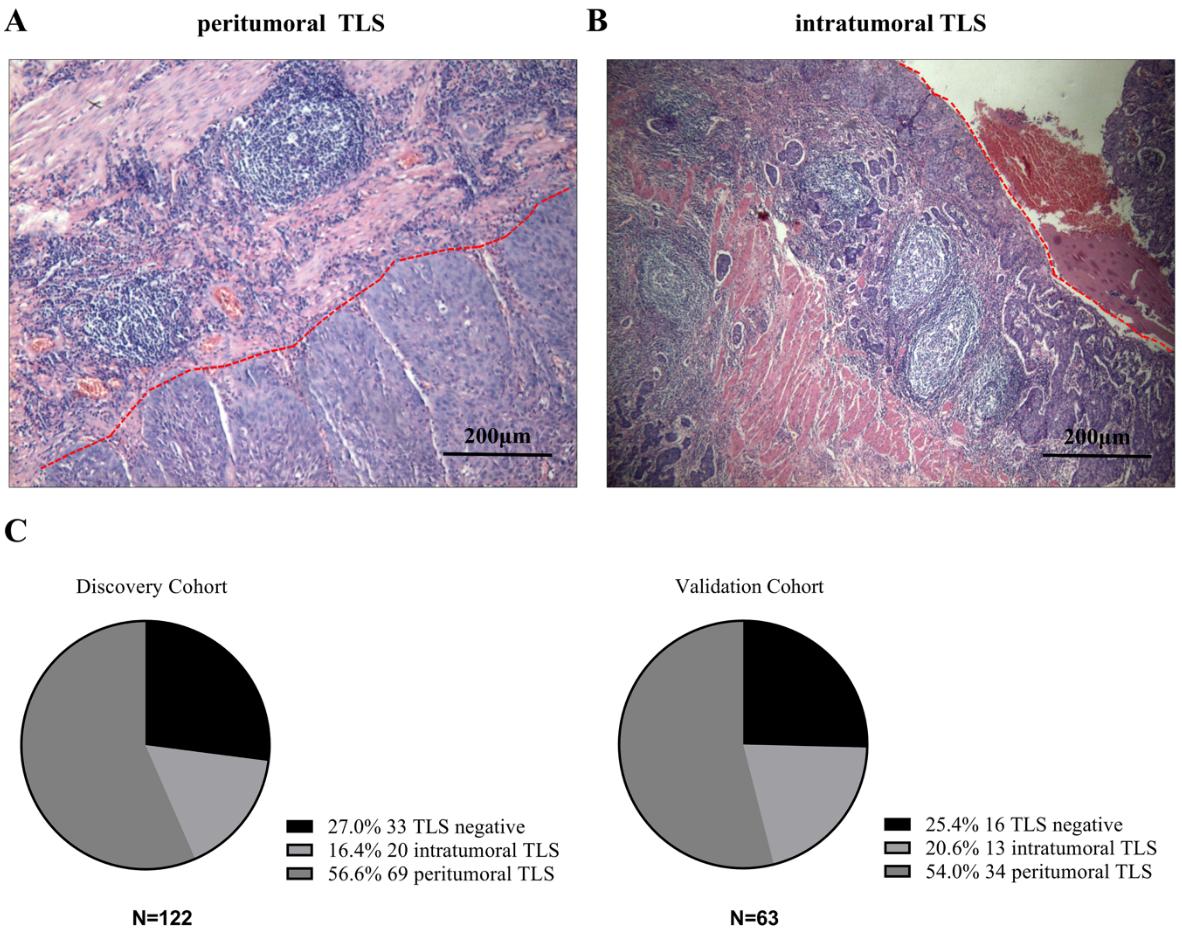

Figure S1. The location of tertiary lymphoid structures (TLSs) in esophageal cancer. (A) Representative H&E images of peritumoral TLS patients. (B) Representative H&E images of intratumoral TLS patients. (C) TLS location evaluated in the discovery (left) and validation cohorts (right). Proportions of TLS negative, peritumoral TLS and intratumoral TLS in ESCC summarized as pie charts.

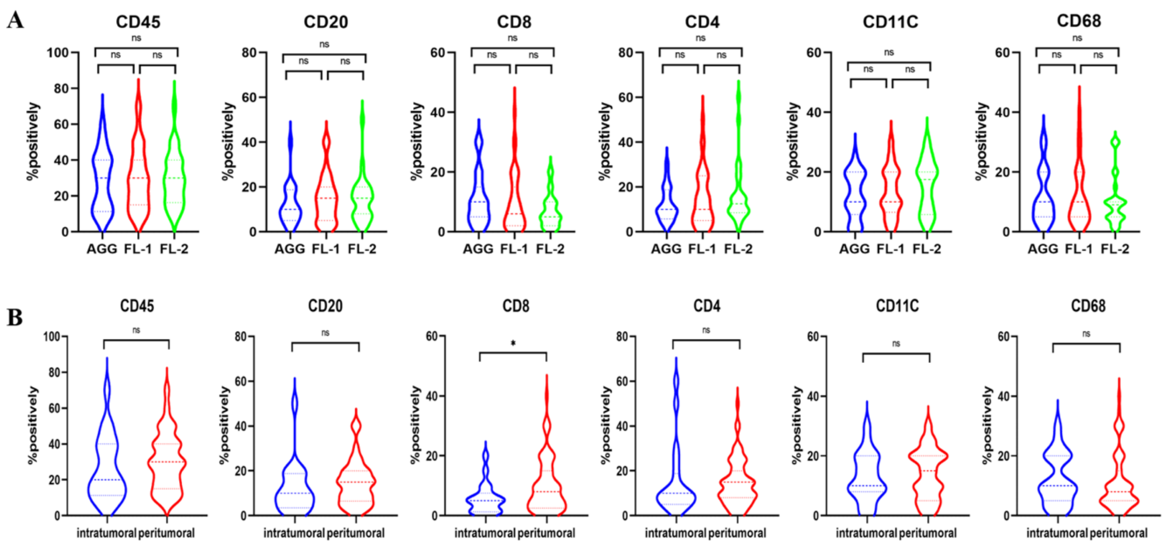

Figure S2. Association between maturation and location of TLSs and immune infiltration. (A) Different CD45 $^{+}$ lymphocytes, CD20^{+} B cells, CD4^{+} T cells, CD8^{+} T cells, CD11c^{+} DCs and CD68^{+} TAMs infiltration between different TLS maturation. (B) Different immune infiltration cells between peritumoral and intratumoral TLS patients.

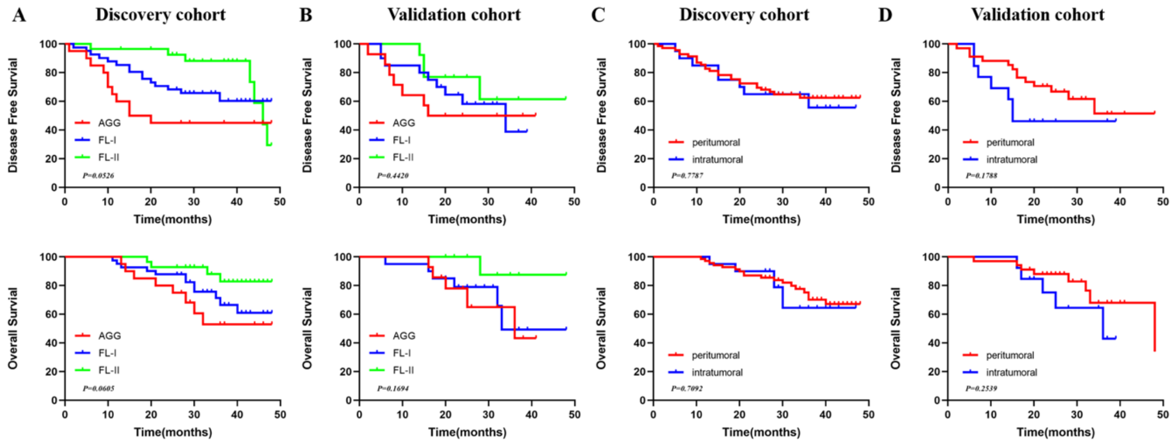

Figure S3. Prognostic significance of TLS maturation in ESCC patients. (A) Kaplan-Meier analysis of Disease-Free Survival (DFS) and overall survival (OS) in the discovery cohort according to TLS phenotypes (n = 122). (B) Kaplan-Meier analysis of DFS and OS in the validation cohort according to TLS phenotypes (n = 63). (C) Kaplan-Meier analysis of DFS and OS in the discovery cohort according to TLS location (n = 122). (D) Kaplan-Meier analysis of DFS and OS in the validation cohort according to TLS location (n = 122)

Table S1. The antibodies and conditions used for IHC in this study

Antibody	CD45	CD20	CD4	CD8	CD11C	CD68
Company	proteintech	proteintech	CST	CST	Abcam	Abcam
Catalog number	60287-1-Ig	60271-1-Ig	#48274	#85336	ab52632	ab283316
Clonality	Monoclonal	Monoclonal	Monoclonal	Monoclonal	Monoclonal	Monoclonal
Clone	4E9B2	4A7G3	EP204	D8A8Y	EP1347Y	EPR20545
Host	Mouse	Mouse	Rabbit	Rabbit	Rabbit	Mouse
Storage	‘-20℃	‘-20℃	‘-20℃	‘-20℃	‘-20℃	‘-20℃
Primary dilution	1:4000	1:1000	1:100	1:200	1:500	1:5000
Dilution solution	Tris/EDTA buffer	Tris/EDTA buffer	SignalStain ® Antibody Diluent	SignalStain ® Antibody Diluent	Tris/EDTA buffer	Citrate buffer
